# Toxicity Profiling of Biosurfactants Produced by Novel Marine Bacterial Strains

**DOI:** 10.3390/ijms22052383

**Published:** 2021-02-27

**Authors:** Georgia-Persephoni Voulgaridou, Theodora Mantso, Ioannis Anestopoulos, Ariel Klavaris, Christina Katzastra, Despoina-Eugenia Kiousi, Marini Mantela, Alex Galanis, Konstantinos Gardikis, Ibrahim M. Banat, Tony Gutierrez, Karina Sałek, Stephen Euston, Mihalis I. Panayiotidis, Aglaia Pappa

**Affiliations:** 1 Department of Molecular Biology & Genetics, Democritus University of Thrace, 68100 Alexandroupolis, Greece; gvoulgar@mbg.duth.gr (G.-P.V.); i.anestopoulos@yahoo.com (I.A.); arielklvrs@hotmail.com (A.K.); christine.katzastra@gmail.com (C.K.); dkiousi@mbg.duth.gr (D.-E.K.); marimant6@hotmail.com (M.M.); agalanis@mbg.duth.gr (A.G.); 2 Department of Applied Sciences, Northumbria University, Newcastle Upon Tyne NE1 8ST, UK; theodora066@hotmail.com; 3 Research and Development Department, APIVITA SA, Industrial Park Markopoulo Mesogaias, 19003 Athens, Greece; gardikis-k@apivita.com; 4 Pharmaceutical Science Research Group, Biomedical Science Research Institute, Ulster University, Coleraine, Northern Ireland BT52 1SA, UK; im.banat@ulster.ac.uk; 5 Institute of Mechanical, Process & Energy Engineering, School of Engineering & Physical Sciences, Heriot-Watt University, Edinburgh EH14 4AS, UK; tony.gutierrez@hw.ac.uk; 6 Institute of Biological Chemistry, Biophysics & Bioengineering, School of Engineering & Physical Sciences, Heriot-Watt University, Edinburgh EH14 4AS, UK; k.salek@hw.ac.uk (K.S.); S.R.Euston@hw.ac.uk (S.E.); 7 The Cyprus Institute of Neurology and Genetics, Department of Cancer Genetics, Therapeutics and Ultrastructural Pathology, Nicosia 2371, Cyprus; 8 The Cyprus School of Molecular Medicine, The Cyprus Institute of Neurology and Genetics, PO Box 23462, Nicosia 1683, Cyprus

**Keywords:** surface active agents, marine biosurfactants, toxicity profiling, antioxidant activity, anti-mutagenic activity, cytotoxicity, *Marinobacter* strains, *Pseudomonas* strains, in vitro skin model, in vitro liver model

## Abstract

Surface active agents (SAAs), currently used in modern industry, are synthetic chemicals produced from non-renewable sources, with potential toxic impacts on humans and the environment. Thus, there is an increased interest for the identification and utilization of natural derived SAAs. As such, the marine environment is considered a promising source of biosurfactants with low toxicity, environmental compatibility, and biodegradation compared to their synthetic counterparts. MARISURF is a Horizon 2020 EU-funded project aiming to identify and functionally characterize SAAs, derived from a unique marine bacterial collection, towards commercial exploitation. Specifically, rhamnolipids produced by *Marinobacter* MCTG107b and *Pseudomonas* MCTG214(3b1) strains were previously identified and characterized while currently their toxicity profile was assessed by utilizing well-established methodologies. Our results showed a lack of cytotoxicity in *in vitro* models of human skin and liver as indicated by alamar blue and propidium iodide assays. Additionally, the use of the single gel electrophoresis assay, under oxidative stress conditions, revealed absence of any significant mutagenic/anti-mutagenic potential. Finally, both 2,2’-azino-bis (3-ethylbenzothiazoline-6-sulphonicacid) (ABTS) and 2,2-diphenyl-1-picrylhydrazyl radical (DPPH) cell-free assays, revealed no significant anti-oxidant capacity for neither of the tested compounds. Consequently, the absence of significant cytotoxicity and/or mutagenicity justifies their commercial exploitation and potential development into industrial end-user applications as natural and environmentally friendly biosurfactants.

## 1. Introduction

Surface active agents attribute their name to their ability to adsorb to gas-liquid, liquid-liquid as well as solid-liquid interfaces [1]. This is possible due to the amphipathic characteristics of SAAs, which possess a hydrophobic tail and a hydrophilic head. Being amphiphilic, these molecules can partition their hydrophilic moiety towards the aqueous phase and their hydrophobic moiety towards the non-soluble phase, thus acting as emulsifiers as well as foaming, dispersion, and wetting agents [2]. Due to these properties, SAAs are extremely important for cosmetic, pharmaceutical, detergent, and food industries [3].

The vast majority of SAAs used in industry today are synthetic, chemical surfactants. However, due to the environmental effects of these non-degradable SAAs, research has been focused on searching for novel, biodegradable, environmental-friendly, and natural-derived SAAs [4]. These molecules are called biosurfactants and are mainly derived from microorganisms, particularly from bacteria, yeasts, and filamentous fungi. They are often characterized by high biodegradability, increased tolerance to temperature and pH variations, low toxicity, high biocompatibility, and enhanced emulsifying properties [5]. Biosurfactants can be both polymeric and non-polymeric compounds and include fatty acids, glycolipids, lipopeptides, liposaccharides, and neutral lipids [6,7,8]. Apart from their ability to reduce surface and interfacial tension, they often have the potential to interact, both specifically and nonspecifically, with biological molecules, most commonly lipid membranes and proteins. Consequently, they exhibit a panel of bioactivities such as antioxidant, anti-microbial, anti-cancer, anti-aging, and anti-inflammatory properties [9,10]. The health promoting properties of biosurfactants are of great importance for cosmeceutical and food industries, as they are often desirable by the consumers and thus can facilitate the promotion of these products.

Over the past few years, a vast number of new biosurfactants have been isolated from the marine environment. Indeed, marine bacteria have emerged as a valuable source of novel SAAs. Interestingly, marine bacteria that have been identified to produce biosurfactants were mostly found in oil spills. This finding is not surprising, considering the ability of biosurfactant-synthetizing bacteria to produce emulsifiers allowing them to use the hydrophobic hydrocarbon oils as alternative food sources [4].

MARISURF EU Horizon 2020 project aimed to identify novel, naturally derived SAAs from a unique marine bacterial collection and functionally characterize them with respect to different desirable commercial properties. Following the initial screening process, two novel bacterial strains, MCTG107b and MCTG214(3b1), were identified to produce rhamnolipids with properties of enhanced surface tension reduction for potential commercial exploitation. The screening, cultivation process, identification, and isolation of these biosurfactants has been described in detail in previous reports by Twigg et al. (2018) [11] and Tripathi et al. (2019) [12]. Strains MCTG107b and MCTG214(3b1) belong to the genus *Marinobacter* and *Pseudomonas*, respectively. Chemical analysis revealed that the biosurfactant produced from strain MCTG107b is a glycolipid and specifically a mixture of rhamnolipid analogues. Similarly, MCTG214(3b1) strain was also found to synthesize a biosurfactant comprised by rhamnolipid congener, mostly di-rhamnolipids. Importantly, both the Pseudomonas sp. MCTG214(3b1) and *Marinobacter* sp. MCTG107b appeared to be non-pathogenic as illustrated via the *Galleria mellonella* infection model [11,12]. 

Rhamnolipids are the most investigated biosurfactants of the glycolipid group [13]. They contain a hydrophilic group, consisting of either one or two rhamnose monosaccharides linked, via a covalent bond, to a hydrophobic moiety, consisting of one or two β-hydroxy fatty acids [11,14]. Rhamnolipids with one and two rhamnose monosaccharides in the hydrophobic group are categorized as mono-rhamnolipids and di-rhamnolipids, respectively. The bacterial cultivation process results in the production of a mixture of different rhamnolipid congeners, differing in the fatty acid saturation level, in the number of rhamnose monosaccharides and in the length of the hydrophobic chain [14]. Several studies reported that plant and bacteria-derived rhamnolipids exhibit anti-oxidant, anti-viral, and anti-cancer properties [15,16,17,18,19,20,21]. 

In this study, we investigated the toxicity profile of the biosurfactants produced by these two novel strains, MCTG107b and MCTG214(3b1). For this, their free-radical scavenging activity was examined via the 2,2’-azino-bis (3-ethylbenzothiazoline-6-sulphonicacid) (ABTS) and 2,2-diphenyl-1-picrylhydrazyl radical (DPPH) assays. Then, we investigated the cytotoxicity profile of the biosurfactants in human skin and liver cell models by the alamar blue and propidium iodide assays and compared them with synthetic industrial surfactants. Finally, the mutagenic and anti-mutagenic potential of the new biosurfactants under oxidative stress conditions were determined by the single cell gel electrophoresis assay. 

## 2. Results

### 2.1. Cell-Free Radical Scavenging Activity of the Two Biosurfactants

The chemical composition of the biosurfactants produced by the novel bacterial strains MCTG107b and MCTG214(3b1) have been previously described. Chemical analysis studies revealed that the MCTG214(3b1) strain synthesizes biosurfactants comprised by rhamnolipid congeners, mostly di-rhamnolipids [11] (Supplementary Materials, Table S1). Similarly, the biosurfactants produced from strain MCTG107b are a mixture of rhamnolipid analogues [12] (Supplementary Materials, Table S2). To investigate the in vitro anti-oxidant properties of the MCTG107b and MCTG214(3b1) derived biosurfactants, established methodologies were utilized based on the determination of total antioxidant capacity (ABTS assay) and ability to scavenge (inhibit) free radical generation (DPPH assay). Overall, none of the examined biosurfactants exhibited significant radical scavenging activity. Consequently, the IC_50_ values of the biosurfactants could not be accurately estimated (Table 1). Specifically, at the highest concentration tested (1 mg/mL), MCTG107b derived biosurfactant exhibited only 9.67 ± 3.27 and 14.84 ± 0.4% inhibition for DPPH and ABTS, respectively (Table 2). Similarly, the MCTG214(3b1) derived biosurfactant accounted for only negligible antioxidant capacity exhibiting 15.46 ± 4.03 and 10.52 ± 1.75% inhibition at the highest concentration (1 mg/mL) for DPPH and ABTS, respectively. In contrast, ascorbic acid, which was used as a positive control, exhibited significant radical scavenging activity and its IC_50_ values were 25.97 ± 0.25 and 29.32 ± 0.17 for DPPH and ABTS, respectively (Table 1). 

### 2.2. Evaluation of In Vitro Cytotoxicity Profile of SAAs

**Figure 1:**
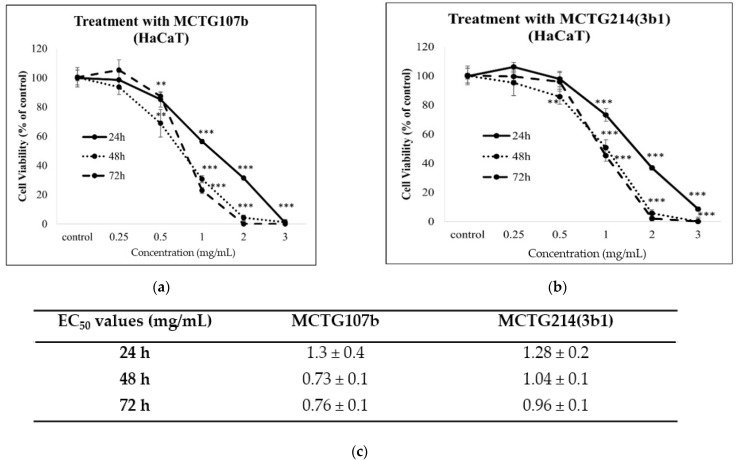
Cytotoxicity profile of MCTG107b (**a**) and MCTG214(3b1) (**b**) derived biosurfactants in the HaCat in vitro skin model. HaCat cells were treated for 24, 48, or 72 h with increasing concentrations (0.25–3 mg/mL) of (**a**) MCTG107b and (**b**) MCTG214(3b1). Table showing EC_50_ values for all incubation periods. (**c**) The viability of cells was determined by utilizing the Alamar blue assay. The results are shown as the mean ± SD and are representative from three independent experiments. Note: ** *p* < 0.01, *** *p* < 0.001 vs. control (untreated cells).

**Figure 2:**
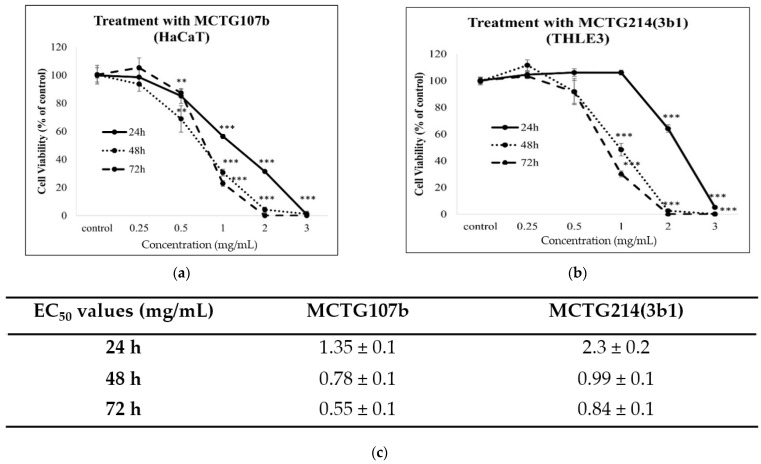
Cytotoxicity profile of MCTG107b (**a**) and MCTG214(3b1) (**b**) derived biosurfactants in the THLE3 in vitro liver model. THLE3 cells were incubated with increasing concentrations (0.25–3 mg/mL) of (**a**) MCTG107b and (**b**) MCTG214(3b1) for 24, 48, or 72 h. Table showing EC_50_ values for all incubation periods. (**c**) The viability of cells was determined by utilizing the alamar blue assay. The results are shown as the mean ± SD and are representative from three independent experiments. Note: *** *p* < 0.001 vs. control (untreated cells).

**Figure 3:**
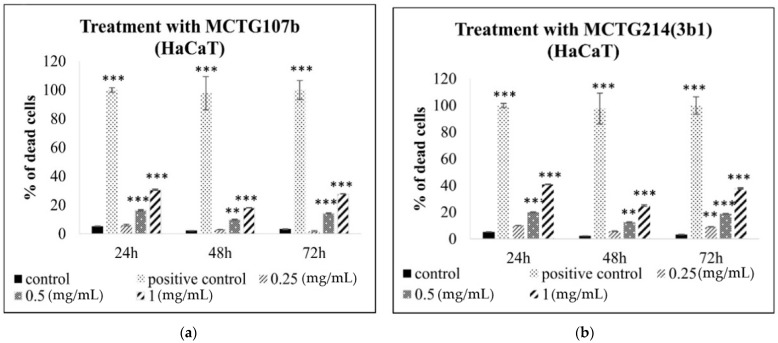

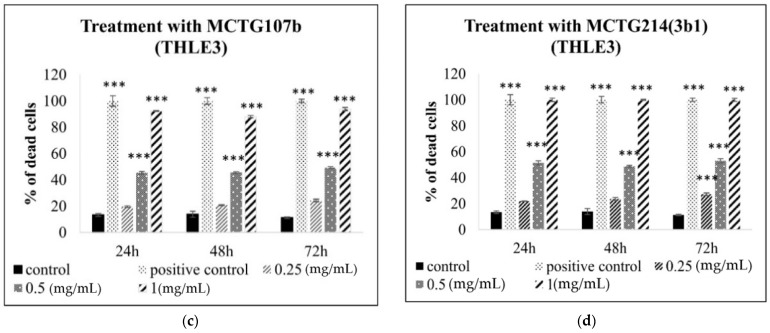
Determination of dead cell populations after exposure to MCTG107b (**a**,**c**) and MCTG214(3b1) (**b**,**d**) derived biosurfactants in the HaCaT and THLE3 in vitro skin and liver models, respectively. Both cell lines were incubated with increasing concentrations (0.25–3 mg/mL) of (**a**,**c**) MCTG107b and (**b**,**d**) MCTG214(3b1) for 24, 48, or 72 h. Levels of dead cells were determined by utilizing the propidium iodide assay. The results are shown as the mean ± SD and are representative from three independent experiments. Note: ** *p* < 0.01, *** *p* < 0.001 vs. control (untreated cells).

Next, alamar blue and propidium iodide assays were used to examine the toxicity profile of the biosurfactants, produced by MCTG107b and MCTG214(3b1), by determining the levels of viable and dead cells in human immortalized keratinocyte (HaCaT; skin) and hepatocyte (THLE-3; liver) cell lines. Levels of viable cells were determined by utilizing the Alamar Blue assay. Cells were cultured in the presence of increasing concentrations (0.25–3 mg/mL) of the biosurfactants for 24, 48, and 72 h. Then the viability of treated cells was determined through the alamar blue assay in comparison to the control-untreated cells. Overall, our data showed that the MCTG107b and MCTG214(3b1) derived biosurfactants exhibit negligible cytotoxicity at concentrations up to 0.25 mg/mL in the skin cell line (Figure 1). However, both biosurfactants were found to be cytotoxic to the HaCaT cells at concentrations beyond 0.25 mg/mL (Figure 1). A similar pattern was observed when utilizing the liver cell line model, as the biosurfactants appeared to be safe at concentrations lower and equal to 0.25 mg/mL (Figure 2). On the contrary, when used at concentrations higher than 0.25 mg/mL, both biosurfactants were highly cytotoxic, with viability being significantly lower than 50% at 1 mg/mL after 48 and 72 h treatments (Figure 2). This observation was also confirmed by the determination of EC_50_ values of both biosurfactants for all incubation periods. More specifically, EC_50_ values for more extended exposure periods (72 h) were lower compared to shorter ones (24 h), whereas it is obvious that THLE3 were more sensitive compared to HaCaT cells with lower EC_50_ values (Figure 1c and Figure 2c). Moreover, we used the propidium iodide assay as another means of determining cytotoxicity by monitoring the levels of dead cells. Similarly, to our previous findings, there was an increase in the rates of dead cell population for both skin and liver models following treatment with 0.25, 0.5, or 1 mg/mL of either biosurfactant (Figure 3). According to our observations, cells incubated with the highest concentration (1 mg/mL) demonstrated the highest elevation in dead cell levels in both cases. Once again, THLE3 were clearly more sensitive (Figure 3c,d) in contrast to HaCaT cells which were considerably more resistant (Figure 3a,b). Based on our results, we selected 0.25 mg/mL as a safe concentration for the subsequent experiments to ensure high cell viability.

**Figure 4:**
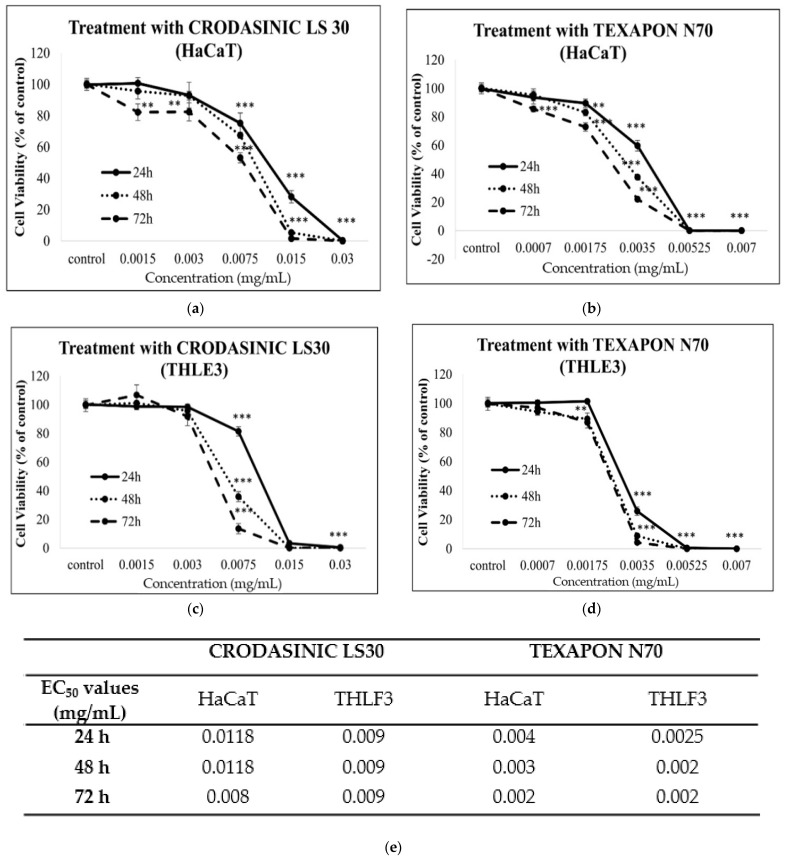
Cytotoxicity profile of Crodasinic LS30 (**a**,**c**) and Texapon N70 (**b**,**d**) surfactants in in vitro skin and liver models. HaCaT and THLE3 cells were incubated with increasing concentrations (0.0015–0.03 mg/mL) of Crodasinic LS30 (**a**,**c**) and (0.0007–0.007 mg/mL) Texapon N70 (**b**,**d**) for 24, 48, or 72 h. Table showing EC_50_ values for all incubation periods (**e**). The viability of cells was determined by utilizing the alamar blue assay. The results are shown as the mean ± SD, and are representative from three independent experiments. Note: ** *p* < 0.01, *** *p* < 0.001 vs. control (untreated cells).

Finally, in order to assess the safety profile of the MCTG107b and MCTG214(3b1) biosurfactants, we exposed HaCaT and THLE3 cells to two synthetic surfactants that are widely used in, for example, cosmetics and pharmaceuticals applications, namely Crodasinic LS30 and Texapon N70. It should be noted that the MCTG107b and MCTG214(3b1) derived biosurfactants (rhamnolipid congeners) do not have similarities in their chemical structure with Crodasinic LS30 and Texapon N70 (Supplementary material, Tables S1 and S2 and Figure S1). Biosurfactants are chemically more complex molecules compared to their synthetic counterparts. Crodasinic LS30 and Texapon N70 are aqueous mixtures of sodium lauroyl sarcosinate (30% *w/v*) (Supplementary Materials, Figure S1a) and sodium lauryl ether sulfate (70% *w/v*) (Supplementary Materials, Figure S1b), respectively. Both cell lines were treated with various concentrations of the surfactant, 0.0015–0.03 mg/mL for Crodasinic LS30 (Figure 4a,c), and 0.0007–0.007 mg/mL for Texapon N70 (Figure 4b,d). Viability was determined using the alamar blue assay (described above) in the case of the biosurfactants for the same incubation periods (24, 48, and 72 h). Our findings indicated that both industrial surfactants were capable of inducing cytotoxicity at much lower concentrations (>0.002 mg/mL) compared to the biosurfactants (>0.5 mg/mL), which is supported by the EC values as well (Figure 4e). These results suggest that the biosurfactants could potentially be a safer alternative for the most common surfactants used currently in industry.

### 2.3. Assessment of Anti-Mutagenic Potential of SAAs In Vitro

**Figure 5:**
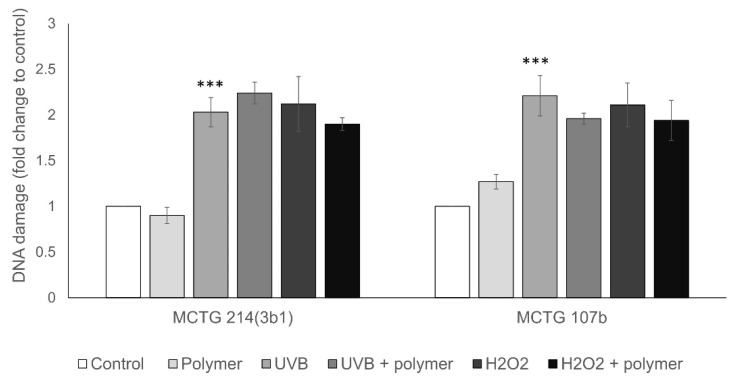
Anti-mutagenic properties of SAAs in an in vitro cancer skin model. A375 cells were pre-incubated with 0.25 mg/mL of MCTG107b/MCTG214(3b1) biosurfactant extract for 48 h and then treated with 5 μM of H_2_O_2_ for 15 min or irradiated with 22 mj/cm^2^ UVB. The levels of DNA damage were subsequently determined through alkaline single cell gel electrophoresis assay. Data represent the mean ± SD fold-change of DNA damage levels compared to the control cells. At least three independent experiments were performed for each condition tested. Note: *** *p* < 0.001 vs. control (untreated cells).

**Figure 6:**
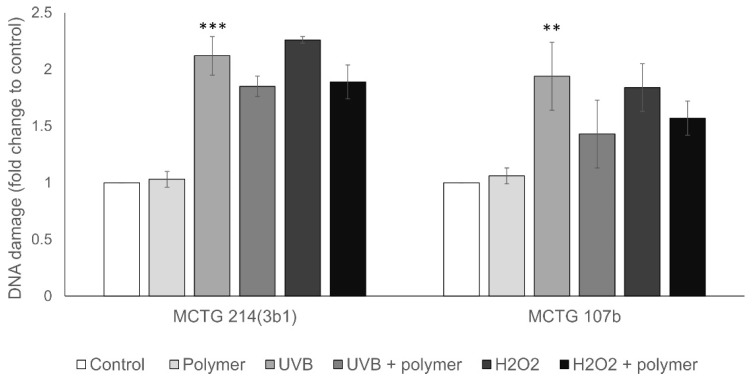
Anti-mutagenic properties of SAAs in an in vitro cancer liver model. Hep-3B cells were pre-incubated for 48 h with 0.25 mg/mL of MCTG107b/MCTG214(3b1) and subsequently irradiated with 27 mj/cm^2^ UVB or treated with 2.5μM of H_2_O_2_ for 15 min. The DNA damage was then determined through alkaline single cell gel electrophoresis assay. Data represent the mean ± SD fold-change of DNA damage levels compared to the control cells. At least three independent experiments were performed for each condition tested. Note: ** *p* < 0.01, *** *p* < 0.001 vs. control (untreated cells).

For evaluating the anti-mutagenic potential of the biosurfactants, the human melanoma A375 and hepatoma Hep3B cell line models have been used. Specifically, A375 and Hep3B cells were pre-incubated with 0.25 mg/mL of biosurfactants for 48 h. Cells were then either treated with H_2_O_2_ (15 min, 5 μM H_2_O_2_ for A375 and 2.5 μM H_2_O_2_ for Hep3B) or irradiated with UVB (22 mJ/cm^2^ for A375 and 27 mJ/cm^2^ for Hep3B) and subsequently DNA damage levels (single strand DNA breaks SSBs) were evaluated by the alkaline single cell gel electrophoresis (comet) assay. UVB and H_2_O_2_ were utilized as two different genotoxic agents, and the specific conditions used for each cell line were chosen to approximately double DNA damage levels. Our results indicate that in A375 cells, the MCTG107b derived biosurfactant slightly reduced both the UVB and H_2_O_2_-induced DNA damage (Figure 5). However, MCTG214(3b1) derived biosurfactants reduced the H_2_O_2_-induced DNA damage, but increased the UVB-induced DNA damage levels (Figure 5). Similar results were, in general, obtained in the case of Hep3B cells, where pre-incubation with both biosurfactants inhibit DNA damage in all conditions tested (Figure 6). Overall, MCTG107b derived biosurfactant exhibited higher geno-protective properties, in comparison to the MCTG214(3b1) produced biosurfactant, however, these effects were statistically non-significant, and thus no anti-mutagenic activity could be established.

## 3. Discussion

Surface acting agents are amphipathic molecules with the ability to adsorb to interfaces of gas-liquid, liquid-liquid, as well as solid-liquid [1]. Due to their unique properties, they have a crucial role in pharmaceutical, detergent, cosmeceutical, and food industry [3]. However, the extensive use of these synthetic, largely non-degradable products comes with several disadvantages, most of which are associated with high environmental risks and/or increased toxicity. Natural-derived biosurfactants constitute an environmentally- friendly, non-toxic alternative that could substitute, to a certain extent, the currently used chemical SAAs [4]. 

Various different microorganisms, mainly species of yeast and bacteria, have been successfully used to produce biosurfactants of various chemical properties. For instance, species of the genera *Bacillus* and *Pseudomonas* are the most common bacterial biosurfactant producers [22]. Nevertheless, their use is restricted, mainly due to their high pathogenicity that makes them inappropriate for use in food related products [23]. In contrast, yeast, such as *Yarrowia lipolytica* and *Saccharomyces cerevisiae*, exhibit a non-pathogenic profile, thus are considered a safer choice for certain applications [5]. 

In the last three decades, the marine environment has emerged as a valuable source for discovering novel biosurfactant-producing microorganisms, including those that produce these biomolecules with novel properties and molecular structures [4]. Specifically, a great number of biosurfactants have been identified from bacterial groups, like *Halomonas, Alcavinorax, Bacillus, Acinetobacter*, and *Pseudomonas* that developed in contaminated waters near oil spills [24,25].

In MARISURF EU Horizon 2020 project, several biosurfactants were identified from a novel collection of marine bacteria and were studied with respect to their surface tension reduction and other properties. From this project, two biosurfactant producing strains were identified, *Marinobacter* sp. strain MCTG107b and *Pseudomonas* sp. strain MCTG214(3b1). The biosurfactants produced by these organisms were identified as rhamnolipid congeners, consisting mainly of di-rhamnolipids, while notably, neither of these *Marinobacter* and *Pseudomonas* strains were found to be pathogenic [11,12]. 

Our goal in this study was to further characterize their bioactive properties and specifically to determine their toxicity profile, as well as to investigate their potential anti-oxidant and anti-mutagenic activities. Our hypothesis, on the potential biological actions of these novel biosurfactants, is based on previous studies reporting that many biosurfactants possess multifunctional activities (e.g., anti-oxidant, anti-cancer, and anti-aging) [9,10]. For instance, He et al. (2016) examined the direct anti-oxidant potential of four different polysaccharides through ABTS assay and reported that all the examined biosurfactants exhibited strong free radical scavenging activity [26]. Similarly, the radical scavenging ability of a biosurfactant derived from the brown seaweed *Sargassum polycystum* was determined via DPPH assay by Palanisamy et al. (2017) [27]. Their results indicated that the biosurfactant showed 61.22% DPPH radical inhibition at 1 mg/mL, while L-ascorbic acid exhibited 2.30% inhibition at the same concentration (1 mg/mL) [27]. 

In our study, we applied a similar methodology to determine the cell-free direct anti-oxidant activity of the two novel biosurfactants. The anti-oxidant properties of the biosurfactants are crucial for food products considering their susceptibility to oxidation, which is responsible for food deterioration [9,28]. Our data indicated that none of the biosurfactants appeared to have a significant free-radical scavenging ability, as indicated by both DPPH and ABTS assays. Previously published data have demonstrated the direct anti-oxidant capacities of certain marine-derived rhamnolipid biosurfactants. Haque et al. (2020) showed that rhamnolipid congeners synthesized by *Marinobacter litoralis* MB15 exhibits 72.6% and approximately 40% DPPH radical scavenging activity at 5 mg/mL and 1 mg/mL concentrations, respectively [15]. The discrepancies observed with our study could be attributed to the different chemical composition of the rhamnolipids analogues. Specifically, the *Marinobacter litoralis* MB15 derived biosurfactant mainly consists of the mono-rhamnolipid congener Rha-C10-C10, while the biosurfactants examined in this study are mainly composed by Rha-Rha-C10-C10, Rha-Rha-C10, and Rha-Rha-C10-C10CH3 analogues [11,12]. 

Abdollahi et al. (2020) [16] also investigated the anti-oxidant capacity of the *Pseudomonas aeruginosa* MN1 produced rhamnolipid biosurfactant via DPPH and ferric reducing antioxidant power (FRAP). The biosurfactant exhibited substantial reducing and free-radical scavenging inhibiting potential, nevertheless the rhamnolipid biosurfactant in this study was examined at concentrations significantly higher than the concentrations of the biosurfactants that we applied [16]. Specifically, the IC_50_ value in DPPH assay for the *Pseudomonas aeruginosa* MN1 derived biosurfactant was 2.277 mg/mL (4.15 mM with a molecular weight of 548.71 Da), whereas the biosurfactants in our study were used at concentrations up to 1 mg/mL, mainly due to insolubility issues. Additionally, the biosurfactant’s major constituent was a Rha-C10-C10 mono-rhamnolipid, so the different compositions among the biosurfactants does not allow the critical comparison between the two studies [16,29].

Another important issue in considering the utilization of biosurfactants in food and cosmetic products is their safety profile. For this reason, we next investigated the cytotoxicity of the novel biosurfactants against the human skin (HaCaT) and the liver (THLE3) cell lines by utilizing the alamar blue and propidium iodide assays. The selection of these models was based on the applications of the biosurfactants in personal care and food industry. Specifically, skin and liver are the main two organs immediately exposed to the biosurfactants following oral administration of a dietary agent or application of a cosmeceutical product. Our results demonstrated that the biosurfactants were safe at concentrations up to 0.25 mg/mL, while they exhibit significant cytotoxicity at concentrations 0.25–3 mg/mL. Few data are available regarding the toxicity profile of rhamnolipid biosurfactants on skin and liver cell models. In particular, a previous report by Haque et al. (2020) describes the toxicity of the *Marinobacter litoralis* MB15 synthesized rhamnolipid biosurfactant against a mouse fibroblast cell model. Briefly, L292 fibroblast cells were treated with 0.05–0.25 mg/mL of the examined biosurfactant for 24 and 48 h, and then the remaining cell viability was determined by the MTT assay. In agreement with our results, rhamnolipid extract demonstrated a safe profile at all conditions tested, as treatment of L292 cells with 0.25 mg/mL for 48 h resulted in 84.7% viability in comparison to the control cells [15].

A number of additional studies also investigate the cytotoxicity profile of rhamnolipid biosurfactants in cancer cell models [20,21,30]. For instance, Rahimi et al. (2019) [30] showed, utilizing the MTT assay, that two *Pseudomonas aeruginosa* MR01 derived rhamnolipid biosurfactants exhibited enhanced antiproliferative activity against breast cancer MCF-7 cells with IC_50_ values of 25.87 μg/mL and 31.00 μg/mL, respectively [30]. Along these lines, *Pseudomonas aeruginosa* B189 rhamnolipid exhibited significant cytotoxicity against MCF-7 cells, with a minimum inhibitory concentration of 6.25 μg/mL [21]. In an attempt to further investigate the potential of the two biosurfactants to be used for industrial purposes as an alternative to current synthetic substances, we selected two widely used surfactants, Crodasinic LS30 and Texapon N70, to compare their effects against the natural derived ones. Crodasinic LS30 is a 30% (*w/v*) mixture of sodium N-lauroylsarcosinate, while Texapon N70 a 70% (*w/v*) mixture of sodium lauryl ether sulfate. Both of them are very well-known anionic surfactants used most commonly in cosmetics, detergents, pharmaceutics, and other products. Our findings showed that both synthetic surfactants appeared to be remarkably more cytotoxic compared to the biosurfactants as their EC_50_ values were much lower. This finding does not come as a surprise considering the general low toxicity of biosurfactants compared to synthetic SAAs [31,32]. This could be explained on the basis that biosurfactants are derived from biological systems, thus increased biological toxicity would have been detrimental for the organisms producing them. Biosurfactants are in general considered promising replacements of synthetic SAAs due to their low toxicity and ecotoxicity, high biodegradability and compatibility with biological systems and their excellent surface active properties [9,33,34]. Sodium lauryl sulfate (SLS), another anionic synthetic surfactant, has been shown to induce the levels of intracellular ROS in HaCaT and reconstructed human epidermal equivalent models by interacting with cellular membranes, therefore suggesting a potent cause for increased skin roughness in response to SLS exposure [35]. Results from a different study demonstrated that SLS caused a higher skin irritation potential (zein value) compared to sodium coco sulfate (SCS) and induced a metabolic reduction in HaCaT cells [36]. Therefore, our study provides further evidence for the promising characteristics of natural derived surfactants and supports their future exploitation. 

Finally, we investigated the potential anti-mutagenic capacity of the novel biosurfactants via single cell gel electrophoresis assay. Importantly, the biosurfactants did not exhibit any mutagenic activity. On the contrary, a pattern indicating minor anti-mutagenic activity was observed in most conditions tested in melanoma (A375) and hepatoma (Hep-3B) cell lines. Nevertheless, this observation is not statistically significant, thus no accurate conclusion can be made at this point. In general, our results are in accordance with previously published studies. For instance, Andreadou et al. (2016) [37] have reported that a *Thermus thermophilus* produced rhamnolipid managed to effectively protect human lymphocytes from the genotoxic effects of camptothecin, as demonstrated by the Sister Chromatin Exchanges assay (SCEs), in a statistically significant manner [37]. However, in contrast to our data, the biosurfactant also exhibited a certain mutagenic activity in the absence of camptothecin [37]. Furthermore, *Pseudomonas putida* derived mono-rhamnolipid biosurfactants exhibited no genotoxicity when tested via the Ames fluctuation assay in a study by Johann et al. [38].

In conclusion, the biosurfactant produced by these novel isolates *Marinobacter* strain MCTG107b and *Pseudomonas* strain MCTG214(3b1), exhibited a relatively safe profile including low cytotoxicity and no genotoxic activity, while no anti-oxidant activity was demonstrated. A slight anti-mutagenic effect was demonstrated, however additional experiments are required to clarify their potential cytoprotective properties. In general, our results were promising in relation with the commercial exploitation of rhamnolipids biosurfactants in food and cosmetic industry.

## 4. Materials and Methods

### 4.1. Materials

The biosurfactants were provided by our project partners from Ulster University as part of the MARISURF EU Horizon 2020 project. The extraction and purification methods were described in detail previously [11,12]. The human cell lines were obtained from the American Type Culture Collection (ATCC, Rockville, MD, USA). Dulbecco’s Modified Eagle’s Medium (DMEM, high glucose), penicillin/streptomycin, fetal bovine serum, and phosphate-buffered saline were obtained from Biosera (Boussens, France). BEGM medium was from Lonza (Lonza/Clonetics Corporation, Walkersville, MD, USA) DPPH, ABTS, and low melting point agarose were purchased from Sigma-Aldrich (BioUltra, Darmstadt, Germany). Resazurin sodium salt and propidium iodide were purchased from Sigma-Aldrich (St. Louis, MO, USA). Crodasinic LS30 and Texapon N70 were supplied by DeWolf Chemical (Warwick, RI, USA).

### 4.2. DPPH and ABTS Assays

ABTS and DPPH assays were carried out as previously described [39]. For DPPH, the extracted biosurfactants were dissolved in methanol to a range of concentrations (0.05–1 mg/mL). A 20 μL volume of each concentration was then added to 180 μL of a 300 μM methanolic solution of DPPH radical for 15 min, at RT in the dark. Twenty μL of the biosurfactant solvent mixed with 180 μL of DPPH radical solution was used as a blank. Subsequently, the absorbance of each sample (at 540 nm) was measured by an ELISA plate reader (EnSpire Multimode Plate Reader, Perkin Elmer, Waltham, MA, USA). The radical scavenging activity was estimated as % inhibition for each condition tested with the following formula:

% DPPH radical scavenging activity = 100 × [(OD_blank_ − OD_sample_)/OD_blank_)]. (1)

For ABTS assay, 2.45 mM potassium persulfate was added to 7 mM ABTS for 16 h, under darkness to induce the formation of the ABTS cation (ABTS+). Then, 180 μL of the ABTS·+ solution were mixed with 20 μL of biosurfactant dilutions (0.05–1 mg/mL), prepared in methanol, for 30 min, at room temperature. As a blank, 20 μL of biosurfactant’s solvent were mixed with 180 μL of the ABTS·+ solution that was used. The absorbance of each sample at 734 nm was determined using an Elisa plate reader (EnSpire Multimode Plate Reader, Perkin Elmer, Waltham, MA, USA) and the antioxidant capacity was estimated as % inhibition of the cation ABTS·+, for each concentration tested, by using the following formula:

% ABTS·+ radical scavenging activity = 100 × [(OD_blank_ − OD_sample_)/OD_blank_)]. (2)

All samples were examined in three replicates. Ascorbic acid, a known antioxidant substance, was used as a positive control for both assays.

### 4.3. Cell Culture and Treatments

Human immortalized keratinocyte HaCaT cell line, melanoma A375 cell line and hepatocellular carcinoma Hep-3B cell line were all maintained in Dulbecco’s Modified Eagle’s Medium high glucose, supplemented with 10% Fetal Bovine Serum, 100 U/mL penicillin, and 100 μg/mL streptomycin, while liver THLE3 cell line was maintained in BEGM medium supplemented with 5 ng/mL EGF, 70 ng/mL Phosphoethanolamine and 10% fetal bovine serum. All cell lines were cultured at 37 °C, in a 5% CO_2_ humidified atmosphere. Cells were grown as monolayers and were sub-cultured when they reached approximately 90% confluency. For all cell treatments, biosurfactants were initially prepared in methanol at a concentration of 400 mg/mL and then further diluted in complete cell culture medium to the respective working concentration.

### 4.4. Cytotoxicity Assays

The cytotoxicity profile of the biosurfactants was assessed through the alamar blue assay and the propidium iodide assay.

For alamar blue assay, the proper number of HaCaT/THLE-3 cells were seeded into 96-well plates and incubated overnight. On the next day, they were treated with a range of concentrations of the biosurfactants (0.25–3 mg/mL) at a final volume of 100 μL for 24, 48, and 72 h. Following the treatments, 10 μL of 1 mg/mL resazurin reagent were added into each well and mixed by gentle shaking. Samples were incubated at 37 °C for 4 h and then the absorbance was recorded at 570 nm and 600 nm (reference wavelength) by using a Spark multimode plate reader (Tecan, Switzerland). The levels of viable cells were estimated, and cell viability was expressed as percentage of control (untreated) cells. Five replicates (n = 5) of each condition were used in each experiment. 

For the propidium iodide assay, the proper number of HaCaT or THLE-3 cells were seeded into 96-well plates, and on the following day they were treated with 0.25, 0.5, and 1 mg/mL of the biosurfactants (prepared in complete culture media) at a final volume of 100 μL. At the end of 24, 48, and 72 h incubation periods, 100 μL of 5 μM propidium iodide (diluted in PBS) was added into each well for 30 min at 37 °C. Fluorescence was then monitored at 530 nm excitation/620 nm emission and the levels of dead cells were determined based on the detected increase in fluorescence values. As a positive control, cells were treated with 0.2% Triton X-100 in complete medium for 15 min to induce cell death by breakage of their cellular membrane. Levels of dead cells were expressed as the percentage of positive control cells (100% of cells were dead). Five replicates (n = 5) of each condition were used in each experiment.

### 4.5. Single Cell Gel Electrophoresis Assay (Comet Assay)

The comet assay was performed as described previously [40]. Briefly, 2 × 105 of A375/Hep3B cells were seeded on 60 mm plates and cultured for 24 h. Then, cells were pre-treated with 0.25 mg/mL of the biosurfactants, prepared in culture media, for 48 h. Subsequently, cells were either irradiated with UVB in PBS (22 mJ/cm^2^ or 27 mJ/cm^2^ for A375 and Hep3B respectively) using a Bio-Link BLX254 Crosslinker (Vilber Lourmat, Marne-la-Vallée, France) or treated for 15 min with H_2_O_2_ (5 μM or 2.5 μM H_2_O2 for A375 and Hep3B, respectively). Cells were then collected though trypsinization, washed with PBS and suspended (2 × 10^4^ cells) into 1 mL low melting point agarose, prepared in PBS. Cell loaded agarose was then placed onto microscope slides precoated with a thin layer of 1% low melting point agarose. Slides were subsequently immersed in lysis solution (1.2 M NaCl, 100 mM Na_2_EDTA, 0.1% sodium lauryl sarcosinate, 0.26 M NaOH, pH∼13) at 4 °C, under darkness. Following an 1 h incubation, slides were washed with rinse solution (0.03 M NaOH, 2 mM Na_2_EDTA, pH ~12.3) at RT for 20 min. Slides were subjected to electrophoresis (in the rinse solution) for 25 min at 13V, and then washed in dH_2_0 and stained with 10 μg/mL propidium iodide for 20 min. Finally, following a final wash with dH_2_0, they were processed for observation on a Nikon ECLIPSE E200 fluorescence microscope. For estimating DNA damage, 100 comets were analyzed for each slide. Each cell was scored as class 0, 1, 2, 3, or 4 on the basis of the DNA damage levels visualized as “tail”. Each cell was assigned with in regard to its class and the total score of 100 comets ranged from 0 (100% comets being in class 0) up to 400 (100% comets of class 4).

### 4.6. Statistical analysis

Statistical analysis of the experiments and graphs were performed with either GraphPad Prism 5 or Sigma Plot Software v.10. Results are expressed as the mean ± SD of at least three independent experiments performed in three or five replicates. Statistical analysis between two samples were performed by two-tailed Student’s t-test. Analysis of two variables among multiple groups was performed with a two-way ANOVA, followed by Tukey’s multiple comparison test. A *p* ≤ 0.05 was considered as statistically significant.

## Figures and Tables

**Table 1:** Radical scavenging activity of the novel biosurfactants by DPPH and ABTS assays. Data are representative of mean values ± SD of the IC_50_ values. Three replicates were used per experiment. Data is representative of at least three independent experiments. As a positive control, ascorbic acid was used.

IC_50_ (μg/mL)		
	DPPH	ABTS
MCTG107b	n.d.	n.d.
MCTG214(3b1)	n.d.	n.d.
Ascorbic acid	25.97 ± 0.25	29.32 ± 0.17

n.d.: Not determined.

**Table 2:** Free radical scavenging activity of the novel biosurfactants at the highest examined concentration using the DPPH and ABTS assays. Data are representative of mean values ± SD of at least three independent experiments. Three replicates were used per experiment.

Inhibition (%) at 1 mg/mL		
	DPPH	ABTS
MCTG107b	9.67 ± 3.27	14.84 ± 0.4
MCTG214(3b1)	15.46 ± 4.03	10.52 ± 1.75

## Data Availability

The data presented in this study are available within the article. Other data that support the findings of this study are available upon request from the corresponding authors.

## Supplementary Materials

The following are available online at https://www.mdpi.com/1422-0067/22/5/2383/s1, Table S1: Rhamnolipid congeners produced by *Pseudomonas* sp. MCTG214(3b1) and their percentage relative abundance, Table S2: Rhamnolipid congeners produced by *Pseudomonas* sp. MCTG107B and their percentage relative abundance, Figure S1: Chemical structures of (a) sodium lauroylsarcosinate and (b) sodium lauryl ether sulphate.
