# Supplementary material for: Toxicity Profiling of Biosurfactants Produced by Novel Marine Bacterial Strains
Source: Int J Mol Sci. 2021 Feb 27;22(5):2383. doi: 10.3390/ijms22052383 (PMC7956851; doi:10.3390/ijms22052383)
Supplement: Supplementary file 1 [file ijms-22-02383-s001.pdf]

Toxicity profiling of biosurfactants produced by novel marine bacterial strains

Georgia-Persephoni Voulgaridou, Theodora Mantso, Ioannis Anestopoulos, Ariel Klavaris, Christina Katzastra, Despoina-Eugenia Kiousi, Marini Mantela, Alex Galanis, Konstantinos Gardikis, Ibrahim M. Banat, Tony Gutierrez, Karina Salek, Stephen Euston, Mihalis I. Panayiotidis and Aglaia Pappa

Supplementary Material

Table 1. Rhamnolipid congeners produced by *Pseudomonas* sp. MCTG214(3b1) and their percentage relative abundance. Data adapted from [11].

Compound	Molecular form	% Relative abundance
Rha-Rha-C_{10}	$C_{22}H_{40}O_{11}$	23.8
Rha-Rha-C_{10}-C_{10}	$C_{32}H_{58}O_{13}$	42.74
Rha-C_{10}-C_{10}/C_8-C_{12}	$C_{26}H_{48}O_9$	12.26
Rha-Rha-C_{10}-C_{12}/C_{12}-C_{10}	$C_{34}H_{62}O_{13}$	9.78
Rha-Rha-C_8-C_{10}/C_{10}-C_8	$C_{30}H_{54}O_{13}$	11.42

Table 2. Rhamnolipid (mono- and di-rhamnolipid) congeners produced by *Pseudomonas* sp. MCTG107B and their percentage relative abundance. Data adapted from [12].

Compound	Molecular form	% Relative abundance
Rha-$C_{14:2}$	$C_{20}H_{34}O_7$	3.18
Rha-C_{10}-C_{12}/Rha-C_{12}-C_{10}	$C_{28}H_{52}O_9$	0.22
Rha-C_{10}-$C_{10:1}$	$C_{26}H_{46}O_9$	0.27
Rha-C_{12}-C_{12}/Rha-C_{10}-C_{14}	$C_{30}H_{56}O_9$	0.94
Rha-Rha-C_8	$C_{20}H_{36}O_{11}$	1.95
Rha-Rha-C_{10}	$C_{22}H_{40}O_{11}$	5.13
Rha-Rha-C_{14}	$C_{26}H_{48}O_{11}$	0.21
Rha-Rha-C_{10}-$C_{10:1}$/Rha-Rha-$C_{10:1}$-C_{10}	$C_{32}H_{56}O_{13}$	2.85
Rha-Rha-C_{10}-C_{10}	$C_{34}H_{58}O_{13}$	52.45
Rha-Rha-C_{10}-$C_{12:1}$	$C_{33}H_{60}O_{13}$	1.06
Rha-Rha-C_{10}-C_{10}-CH_3	$C_{42}H_{60}O_{13}$	23.07
Decenoyl-Rha-Rha-C_{10}-$C_{10:1}$	$C_{35}H_{72}O_{11}$	0.40
Rha-Rha-C_{10}-C_{12}/Rha-Rha-C_{12}-C_{10}	$C_{35}H_{64}O_{13}$	5.01
Rha-Rha-C_{10}-C_{12}-CH_3/Rha-Rha-C_{12}-C_{10}-CH_3	$C_{35}H_{64}O_{13}$	3.26

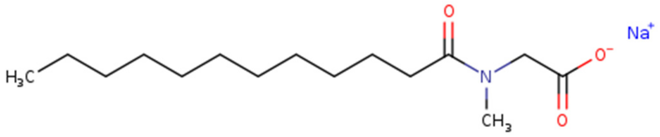

Sodium lauroylsarcosinate

(a)

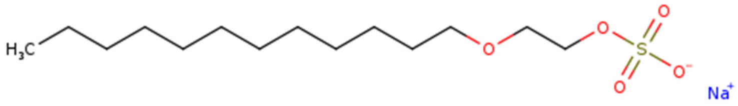

Sodium lauryl ether sulphate

(b)

Figure 1. Chemical structures of (a) sodium lauroylsarcosinate and (b) sodium lauryl ether sulphate.
